# Supplementary material for: Comprehensive Annotation and Functional Exploration of MicroRNAs in Lettuce
Source: Front Plant Sci. 2021 Dec 24;12:781836. doi: 10.3389/fpls.2021.781836 (PMC8739914; doi:10.3389/fpls.2021.781836)
Supplement: Supplementary file 3 [file Data_Sheet_9.ZIP › GSM2230751.plot/Lsa-miRN48_Lsat_1_v5_gn_8_86100.1_266_TPlot.pdf]

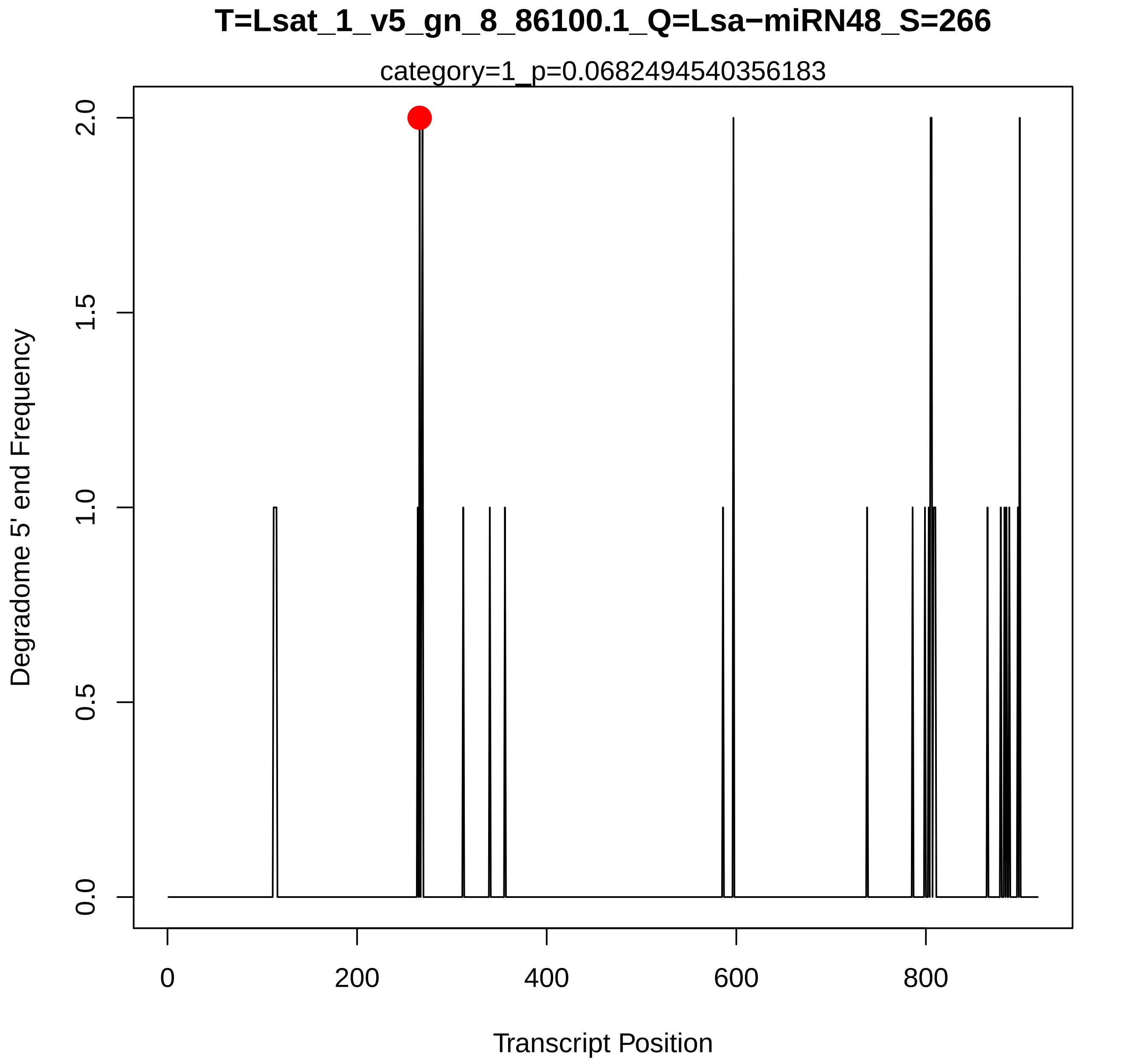

T=Lsat_1_v5_gn_8_86100.1_Q=Lsa−miRN48_S=266
category=1_p=0.0682494540356183
Degradome 5' end Frequency
0.0
0.5
1.0
1.5
2.0
0
200
400
600
800
Transcript Position
